# Bilateral Testicular Tumors Recurred 5 Years After Surgery for an Extragonadal Germ Cell Tumor: Case Report

**DOI:** 10.1002/iju5.70190

**Published:** 2026-05-04

**Authors:** Hiraku Yamamoto, Isao Hara, Satoshi Muraoka, Takahito Wakaymiya, Shimpei Yamashita, Yasuo Kohjimoto

**Affiliations:** ^1^ Department of Urology Wakayama Medical University Wakayama Japan; ^2^ Department of Urology Hashimoto Municipal Hospital Hashimoto Japan

**Keywords:** extragonadal germ cell tumor, seminoma, teratoma, testicular disease, testicular tumor

## Abstract

**Introduction:**

Extragonadal germ cell tumors (EGCT) account for approximately 3%–7% of all germ cell tumors. Metachronous testicular tumors develop in 5%–7% of patients after EGCT treatment; however, bilateral testicular tumors have not been reported.

**Case Presentation:**

A 30‐year‐old man underwent retroperitoneal tumor resection and was diagnosed as teratoma. A cystic lesion in the right testis without malignant features was managed by surveillance. Five years later, he presented with left testicular discomfort. Imaging revealed bilateral testicular tumors, and serum hCG was elevated (11.9 mIU/mL). Bilateral radical orchiectomy revealed pure seminoma (pT1) in the left testis and mixed germ cell tumor with seminoma and teratoma components (pT1) in the right testis.

**Conclusions:**

This is the first reported case of bilateral testicular tumors developing after EGCT treatment. In patients with EGCT, the potential presence of testicular germ cell neoplasia in situ should be considered, and long‐term follow‐up is recommended.

## Keynote Message

We report the first case of bilateral testicular tumors occurring after treatment for EGCT. Careful long‐term surveillance is essential because metachronous tumors may develop even several years after initial therapy.

## 1 Introduction

Extragonadal germ cell tumors (EGCT) are rare, accounting for approximately 3%–7% of all germ cell tumors. Testicular tumors may develop during follow‐up after treatment for EGCT, with a reported incidence of 5%–7%. However, bilateral testicular tumor development following EGCT has not previously been described. We report a rare case of bilateral testicular tumors detected 5 years after surgical treatment for EGCT.

## 2 Case Presentation

**FIGURE 1:**
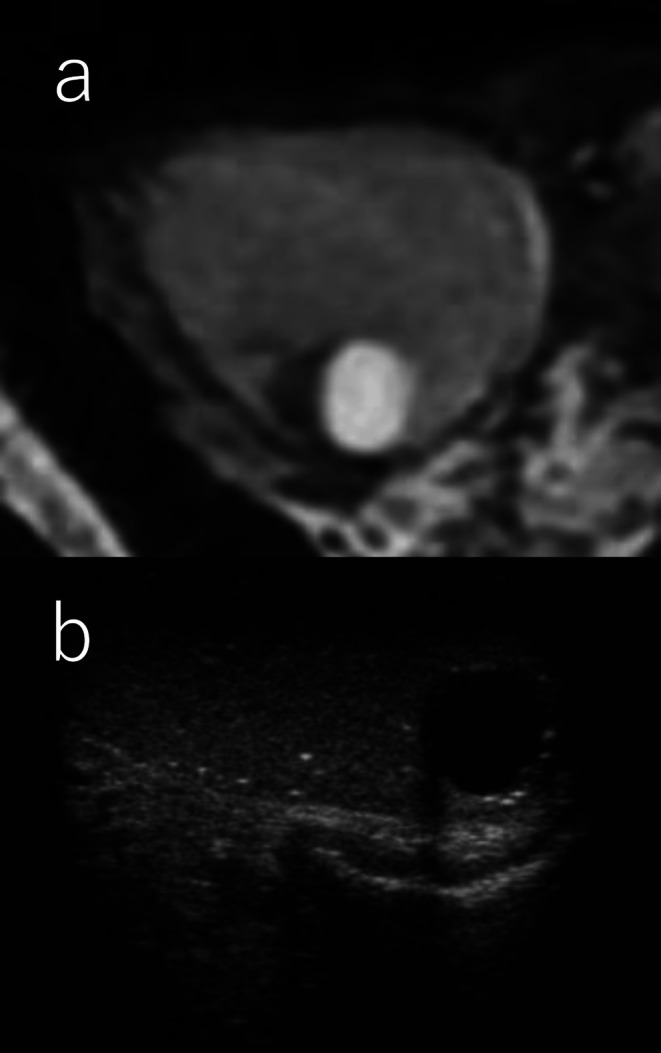
Magnetic Resonance Imaging and Ultrasonograms of bilateral testes at initial presentation. (a, b) Magnetic resonance imaging (MRI) and Ultra sounds (US) revealed only a 10‐mm simple cystic lesion in the right testis.

A 30‐year‐old man presented with backache. Computed tomography revealed a retroperitoneal tumor and a 10 mm cystic lesion in the right testis. Serum tumor markers, including hCG, AFP, and LDH, were within normal ranges. Ultrasonography and magnetic resonance imaging (MRI) showed no solid or malignant features in the right testis (Figure 1).

Following the diagnosis of EGCT, a retroperitoneal tumor was resected, which was pathologically diagnosed as teratoma. Although metastasis from occult testicular germ cell neoplasia in situ (GCNIS) or a burned‐out tumor could not be definitively excluded, testicular biopsy was not performed because the development of metachronous testicular tumors is relatively low even when GCNIS is present, and prognosis remains favorable even if secondary tumors develop. Adjuvant chemotherapy was also not administered because tumor markers were negative and the resected tumor was a pure teratoma, which is generally resistant to chemotherapy. During follow up, the imaging findings of the right testicular cystic lesion and tumor markers remained stable. Four years after surgery, regular follow‐up was interrupted due to the COVID‐19 pandemic.

**FIGURE 2:**
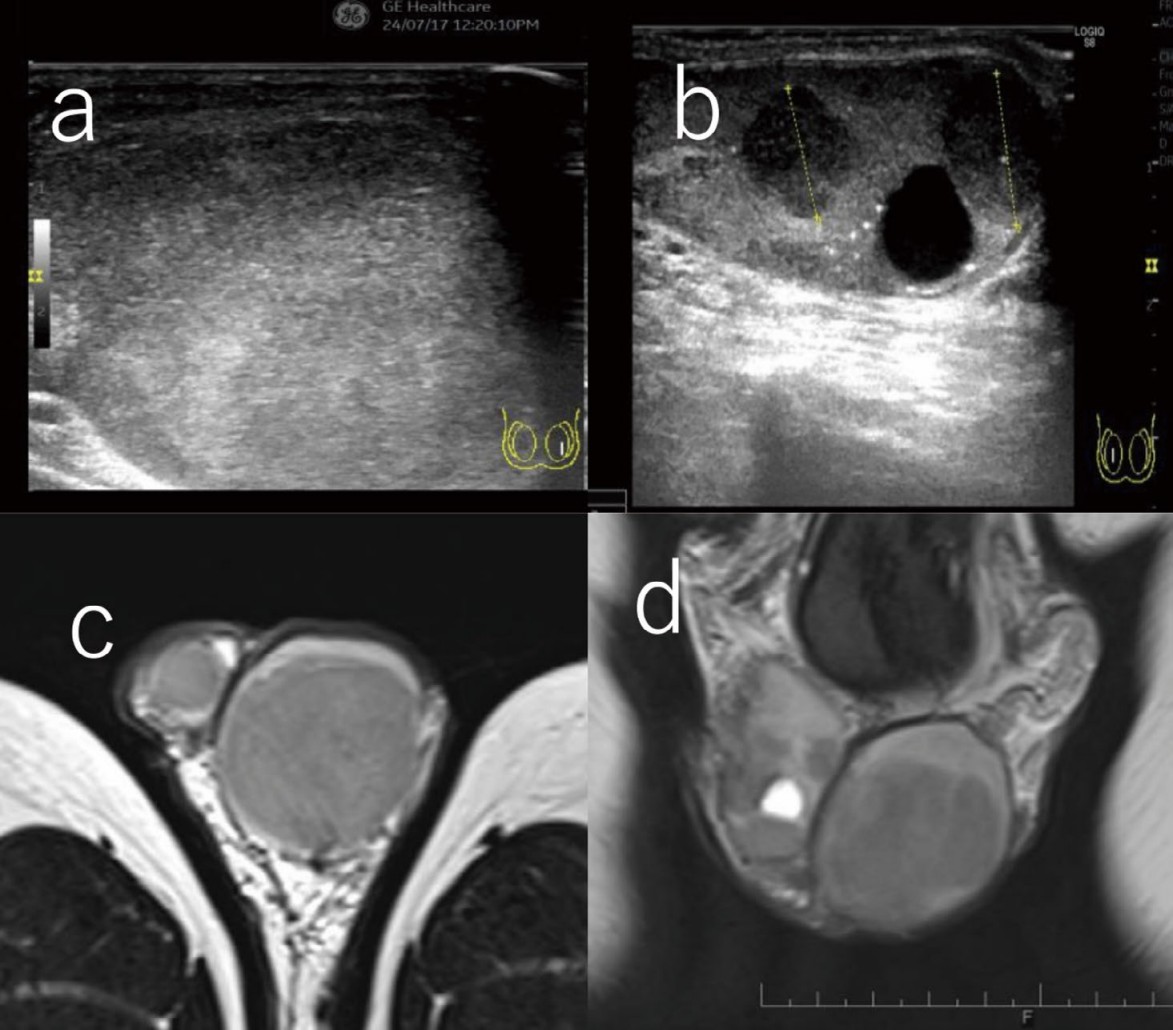
MRI and US of bilateral testes after 5 years: (a) The left testis was entirely mosaic‐like and replaced by a neoplastic lesion. (b) Low echoing areas 9.7 mm and 11.1 mm in diameter were observed on a cystic nodular lesion. (c, d) MRI showed a low‐signal neoplastic lesion in the left testis on T2‐weighted imaging, and a similar neoplastic lesion around a cystic lesion in the right testis.

Five years after the initial surgery, the patient returned with a two‐month history of discomfort in the left testis. Physical examination revealed enlargement and stony hardness of the left testis. Ultrasonography demonstrated complete replacement of the left testis by a heterogeneous neoplastic lesion. In the right testis, in addition to the pre‐existing cystic lesion, new hypoechoic solid lesions measuring 9.7 mm and 11.1 mm were identified (Figure 2a,b). Serum hCG was elevated to 11.9 mIU/mL.

**FIGURE 3:**
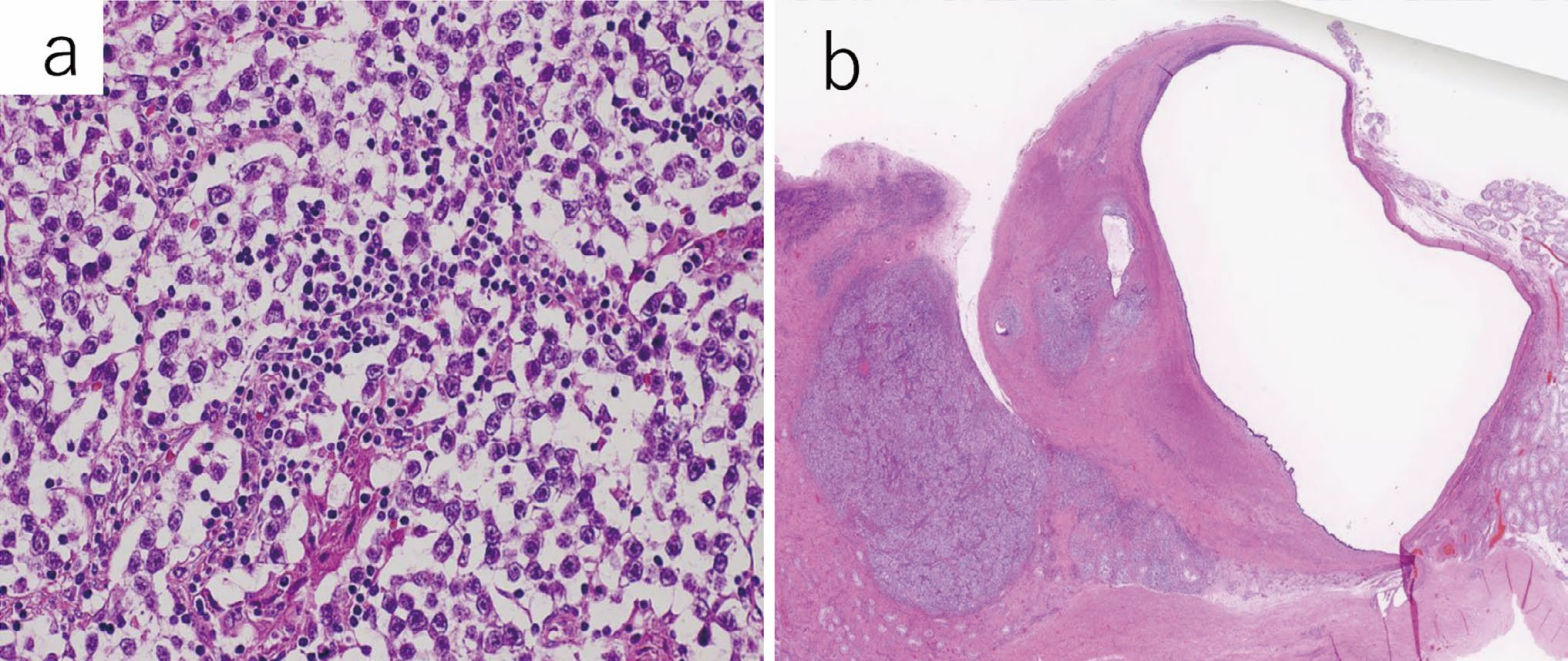
Microscopic findings of bilateral testes. The pathological diagnosis was pure seminoma, pT1, in the left testis (a), and mixed germ cell tumor with seminoma and teratoma components, pT1, in the right testis (b).

MRI showed low‐signal intensity tumors in both testes on T2‐weighted imaging (Figure 2c,d). Bilateral testicular tumors were diagnosed, and bilateral radical orchiectomy was performed. Pathological examination revealed pure seminoma (pT1) in the left testis and mixed germ cell tumor consisting of seminoma and teratoma components (pT1) in the right testis (Figure 3).

## 3 Discussion

EGCT accounts for 3%–7% of all germ cell tumors, with retroperitoneal primary cases comprising approximately 30%–40% of them. The form of occurrence is classified as either primary or secondary, with the latter considered a metastatic lesion originating from germ cell neoplasia in situ (GCNIS) present in the testis or a burned‐out tumor.

Additionally, patients with EGCT often have concurrent testicular GCNIS. Regarding its prevalence, a study involving 68 patients with EGCT who underwent testicular biopsy before starting treatment reported that GCNIS was detected in 21 cases (31%), and four patients developed metachronous testicular tumors (MTT) [1]. However, there are also negative opinions regarding testicular biopsy in EGCT patients because the incidence of MTT is not particularly high and prognosis remains favorable with appropriate treatment, even if MTT develops.

In this case, 1 cm cystic lesion was noted in the right testis. However, MRI and ultrasound examinations revealed no solid components, leading us to consider the retroperitoneal tumor as metastasis from a testicular tumor unlikely. Nevertheless, since the possibility of retroperitoneal tumor metastasis from testicular GCNIS or a burned‐out tumor could not be ruled out, we discussed the feasibility of testicular biopsy. Based on the reasons outlined above, we decided not to perform biopsy but to follow up under strict surveillance. Regarding adjuvant chemotherapy, it was deemed unnecessary because all tumor marker values were below the reference range and the excised pathological specimen was a pure teratoma which was highly resistant to chemotherapy. However, since GCNIS is highly sensitive to chemotherapy, greater vigilance regarding the development of synchronous or metachronous testicular tumors should have been kept in mind when choosing surveillance.

Here, we presented a case of EGCT that relapsed in both testes after treatment.

The frequency of testicular recurrence following EGCT treatment has been reported to be approximately 5% [2]. A literature review identified 32 reported cases, including our patient (Table 1). The patients were 18–49, with a median age of 30 years.

**TABLE 1:** Reported cases of testicular recurrence after EGCT treatment.

No.	Author	Age	Pathology of EGCT	Pathology of MTT		Interval
1	Quintela [3]	44	Sem	Non‐sem	Unilateral	84
2	Lokich [4]	22	Non‐sem	Sem	Unilateral	168
3	Hayashi [5]	18	Non‐sem	Sem	Unilateral	84
4	Gerl [6]	Unknown	Non‐sem	Sem	Unilateral	35
5	Gerl [6]	Unknown	Non‐sem	Sem	Unilateral	42
6	Gerl [6]	Unknown	Non‐sem	Sem	Unilateral	77
7	Allaway [7]	22	Non‐sem	Sem	Unilateral	84
8	Daniel [8]	24	Sem	Non‐sem	Unilateral	60
9	Daniel [8]	23	Non‐sem	Non‐sem	Unilateral	23
10	Hartmann [2]	28	Non‐sem	Sem	Unilateral	74
11	Hartmann [2]	33	Non‐sem	Non‐sem	Unilateral	30
12	Hartmann [2]	40	Non‐sem	Sem	Unilateral	100
13	Hartmann [2]	49	Non‐sem	Sem	Unilateral	88
14	Hartmann [2]	29	Non‐sem	Non‐sem	Unilateral	30
15	Hartmann [2]	22	Non‐sem	Sem	Unilateral	48
16	Hartmann [2]	23	Non‐sem	Sem	Unilateral	14
17	Hartmann [2]	34	Non‐sem	Sem	Unilateral	35
18	Hartmann [2]	Unknown	Non‐sem	Sem	Unilateral	102
19	Hartmann [2]	30	Non‐sem	Sem	Unilateral	42
20	Hartmann [2]	34	Non‐sem	Sem	Unilateral	78
21	Hartmann [2]	22	Non‐sem	Non‐sem	Unilateral	36
22	Mindrup [9]	42	Non‐sem	Non‐sem	Unilateral	50
23	Kuroda [10]	32	Non‐sem	Sem	Unilateral	48
24	Yamada [11]	30	Unknown	Sem	Unilateral	96
25	Kawamura [12]	33	Unknown	Non‐sem	Unilateral	91
26	Hashimoto [13]	27	Non‐sem	Sem	Unilateral	120
27	Hashimoto [13]	30	Sem	Sem	Unilateral	96
28	Hashimoto [13]	38	Non‐sem	Non‐sem	Unilateral	64
29	Hashimoto [13]	32	Non‐sem	Sem	Unilateral	15
30	Hashimoto [13]	47	Non‐sem	Non‐sem	Unilateral	21
31	Tobiume [14]	49	Non‐sem	Sem	Unilateral	248
32	Present case	30	Non‐sem	Sem	Bilateral	59
Median		30				62

Abbreviations: EGCT, extraconadal germ cell tumor; MTT, metachronous testicular tumor.

Regarding histology, 27 out of 32 cases (84%) of EGCT were non‐seminomas, whereas 22 out of 32 cases (69%) of MTT were seminomas. The time to MTT occurrence ranged between 14 and 248 months, with a median of 62 months.

The reason why EGCT has more non‐seminoma and MTT has more seminoma is thought to be due to differences in the time elapsed since differentiation from GCNIS. According to one widely‐accepted theory, GCNIS reportedly acquires invasiveness and differentiates into seminoma, and then that seminoma further differentiates into non‐seminoma [15]. In EGCT, lesions develop deep within the body, often requiring a longer time for detection. In contrast, testicular tumors are more likely to be identified at an earlier stage due to scrotal swelling, which allows diagnosis at the seminoma stage.

We are skeptical that the seminoma tumor in the left testis was a metastasis from the right testicular tumor. In cases where a contralateral testicular tumor arises as part of systemic hematogenous spread or extensive lymphatic metastasis, it is natural to classify it as metastatic. However, in this case, no metastatic lesions were observed outside the testes.

Secondly, if the left testicular tumor was indeed a metastasis from the right testicular tumor, it would imply that a non‐seminoma had reverted to a seminoma at the metastatic site, which contradicts the reported theory of tumor progression [15].

We therefore suggest that even if a high inguinal orchiectomy of the right testis had been performed at an earlier stage, it is unlikely that the development of a contralateral testicular tumor could have been prevented in this case.

In our case, there was unfortunately a period during the pandemic of the patient being lost to follow‐up, and this ultimately necessitated bilateral orchiectomy. With unbroken careful follow‐up, management of the left testicular tumor with partial rather than radical orchiectomy might have been possible. We suggest that testicular lesions following EGCT require meticulous long‐term follow‐up to ensure timely detection and appropriate management.

## 4 Conclusion

In patients with EGCT, the possibility of metachronous tumors originating from testicular GCNIS should be considered. Long‐term follow‐up is essential, along with patient education on recurrence risk, proper self‐examination techniques, and other preventive measures.

## Consent

Written informed consent was obtained from the patient.

## Conflicts of Interest

Yasuo Kohjimoto is an Editorial Board member of *International Journal of Urology Case Reports* and a co‐author of this article. To minimize bias, he was excluded from all editorial decision‐making related to the acceptance of this article for publication.

## Data Availability

The data that support the findings of this study are available from the corresponding author upon reasonable request.

## References

[1] S. D. Fosså , N. Aass , A. Heilo , et al., “Testicular Carcinoma In Situ in Patients With Extragonadal Germ‐Cell Tumours: The Clinical Role of Pretreatment Biopsy,” Annals of Oncology 14, no. 9 (2003): 1412–1418, 10.1093/annonc/mdg373. 12954581

[2] J. T. Hartmann , S. D. Fossa , C. R. Nichols , et al., “Incidence of Metachronous Testicular Cancer in Patients With Extragonadal Germ Cell Tumors,” Journal of the National Cancer Institute 93, no. 22 (2001): 1733–1738, 10.1093/jnci/93.22.1733. 11717334

[3] A. González Quintela , E. López Bonet , J. Román , and P. Aramburo , “Testicular Germ Cell Tumor Seven Years After a Retroperitoneal Germ Cell Tumor,” European Urology 19, no. 4 (1991): 336–338, 10.1159/000473655. 1655464

[4] J. Lokich , “Metachronous Gonadal and Extragonadal Primary Germ Cell Tumors: Two Case Reports,” Cancer Investigation 12, no. 4 (1994): 406–408, 10.3109/07357909409038232. 8032962

[5] T. Hayashi , M. Mine , S. Kojima , and H. Sekine , “Extragonadal Germ Cell Tumor Followed by Metachronous Testicular Tumor. A Case Report,” Urologia Internationalis 57, no. 3 (1996): 194–196, 10.1159/000282911. 8912452

[6] A. Gerl , C. Clemm , R. Lamerz , and W. Wilmanns , “Cisplatin‐Based Chemotherapy of Primary Extragonadal Germ Cell Tumors,” A Single Institution Experience. Cancer 77, no. 3 (1996): 526–532, 10.1002/(SICI)1097-0142(19960201)77:3<526::AID-CNCR15>3.0.CO;2-6. 8630961

[7] M. Allaway and U. O. Nseyo , “Primary Testicular Seminoma in a Patient With a History of Extragonadal Non‐Seminomatous Germ Cell Carcinoma,” Urology 55, no. 6 (2000): 949–950, 10.1016/s0090-4295(99)00614-7. 10840119

[8] C. Daniel , K. Fizazi , S. Culine , L. Zelek , P. Wibault , and C. Theodore , “Metachronous Gonadal and Extragonadal Primaries, or Late Relapse of Germ Cell Tumor?,” Urologic Oncology 6, no. 2 (2001): 49–52, 10.1016/s1078-1439(00)00091-0. 11166620

[9] S. R. Mindrup and B. R. Konety , “Testicular Recurrence From ‘Primary’ Retroperitoneal Germ Cell Tumor,” Urology 64, no. 5 (2004): 1031, 10.1016/j.urology.2004.06.016. 15533510

[10] I. Kuroda , M. Ueno , T. Mitsuhashi , et al., “Testicular Seminoma After the Complete Remission of Extragonadal Yolk Sac Tumor: A Case Report,” BMC Urology 4 (2004): 13, 10.1186/1471-2490-4-13. 15546481 PMC535804

[11] Y. Yamada , K. Tomita , T. Fujimura , H. Nishimatsu , T. Takeuchi , and T. Kitamura , “Metachronous Testicular Tumor Developing Eight Years After Retroperitoneal Extragonadal Germ Cell Tumor,” International Journal of Urology 15, no. 3 (2008): 267–269, 10.1111/j.1442-2042.2007.01971.x. 18304228

[12] N. Kawamura , K. Yamamoto , I. Yoshioka , et al., “A Case of Metachronous Testicular Tumor Developing Seven Years After Complete Remission of Retroperitoneal Extragonadal Germ Cell Tumor,” Hinyokika Kiyo 55 (2009): 635–638. 19926951

[13] K. Hashimoto , H. Fujimoto , T. Kouno , et al., “The Incidence and Management of Metachronous Testicular Germ Cell Tumors in Patients With Extragonadal Germ Cell Tumors,” Urologic Oncology 30, no. 3 (2012): 319–324, 10.1016/j.urolonc.2010.02.008. 20471872

[14] M. Tobiume , S. Aoki , G. Nishikawa , et al., “Late Testicular Relapse Two Decades After Primary Extragonadal Germ Cell Tumor With Uncommon Metastases: A Case Report,” Journal of Medical Case Reports 15 (2021): 59, 10.1186/s13256-021-02667-y. 33541424 PMC7863479

[15] J. W. Oosterhuis and L. H. Looijenga , “Current Views on the Pathogenesis of Testicular Germ Cell Tumours and Perspectives for Future Research: Highlights of the 5th Copenhagen Workshop on Carcinoma In Situ and Cancer of the Testis,” APMIS 111 (2003): 280–289, 10.1034/j.1600-0463.2003.1110131.x. 12752274

